# Inhibitory activity of *Lactobacillus plantarum* LMG P-26358 against *Listeria innocua* when used as an adjunct starter in the manufacture of cheese

**DOI:** 10.1186/1475-2859-10-S1-S7

**Published:** 2011-08-30

**Authors:** Susan Mills, L Mariela Serrano, Carmel Griffin, Paula M O'Connor, Gwenda Schaad, Chris Bruining, Colin Hill, R Paul Ross, Wilco C Meijer

**Affiliations:** 1 Teagasc Food Research, Moorepark, Fermoy, Co. Cork, Ireland; 2 Alimentary Pharmabiotic Centre, Cork, Ireland; 3 CSK Food Enrichment, Ede, The Netherlands; 4 Department of Microbiology, University College Cork, Ireland

## Abstract

*Lactobacillus plantarum* LMG P-26358 isolated from a soft French artisanal cheese produces a potent class IIa bacteriocin with 100% homology to plantaricin 423 and bacteriocidal activity against *Listeria innocua* and *Listeria monocytogenes*. The bacteriocin was found to be highly stable at temperatures as high as 100°C and pH ranges from 1-10. While this relatively narrow spectrum bacteriocin also exhibited antimicrobial activity against species of enterococci, it did not inhibit dairy starters including lactococci and lactobacilli when tested by well diffusion assay (WDA). In order to test the suitability of *Lb. plantarum* LMG P-26358 as an anti-listerial adjunct with nisin-producing lactococci, laboratory-scale cheeses were manufactured. Results indicated that combining *Lb. plantarum* LMG P-26358 (at 10^8^ colony forming units (cfu)/ml) with a nisin producer is an effective strategy to eliminate the biological indicator strain, *L. innocua*. Moreover, industrial-scale cheeses also demonstrated that *Lb. plantarum* LMG P-26358 was much more effective than the nisin producer alone for protection against the indicator. MALDI-TOF mass spectrometry confirmed the presence of plantaricin 423 and nisin in the appropriate cheeses over an 18 week ripening period. A spray-dried fermentate of *Lb. plantarum* LMG P-26358 also demonstrated potent anti-listerial activity *in vitro* using *L. innocua*. Overall, the results suggest that *Lb. plantarum* LMG P-26358 is a suitable adjunct for use with nisin-producing cultures to improve the safety and quality of dairy products.

## Introduction

Bacteriocins are ribosomally synthesized, heat stable, antimicrobial peptides which are produced by a wide variety of bacteria. These peptides can have a narrow spectrum of inhibition (inhibiting closely related bacteria) or a broad spectrum (inhibiting a wide range of bacteria) [1]. The bacteriocins of lactic acid bacteria (LAB) have been the topic of much interest in terms of food safety due to their “generally recognised as safe” (GRAS) status [2]. They have been classified into as many as three different groups based on their biochemical and genetic properties [3]. For example, the well known bacteriocin nisin is a member of the class I group of bacteriocins. These peptides are also referred to as lantibiotics as they contain unusual amino acid derivatives such as lanthionine and/or β-methylanthionine as well as other post-translational modifications. Class II contains unmodified peptides and are further subdivided into three subclasses; class IIa (one-peptide pediocin-like bacteriocins), class IIb (two-peptide bacteriocins), and class IIc (other non-pediocin-like one-peptide bacteriocins), while class III peptides consist of the thermosensitive proteins [4].

	In an intensive screening program for potential anti-listerial cheese starters, a potent bacteriocin producer was isolated from a French artisanal soft cheese. The producing strain was identified as *Lactobacillus plantarum* by 16S rDNA sequencing and was later designated *Lb. plantarum* LMG P-26358. The strain produces a proteinaceous compound with activity against *Listeria innocua* and *Listeria monocytogenes*. Sequence analysis indicated that the bacteriocin was a member of the class IIa group of bacteriocins, which are recognised for their potent activity against *Listeria*. Moreover, this group of peptides can be defined by a highly conserved charged N-terminal domain with the consensus sequence YGNGV(X)C(X)_4_C(X)V(X)_4_A (where X denotes any amino acid) and a more variable hydrophobic and/or amphiphilic C-terminal domain [3]. Structurally, these peptides contain an N-terminal β-sheet-like domain which is stabilised by a conserved disulfide bridge and a C-terminal domain consisting of one or two α-helices, which can end with a structurally extended C-terminal tail [5]. A few class IIa bacteriocins have also been shown to contain a second disulfide bridge in the C-terminal region. This extra bridge has been associated with increased stability of the 3D structure [5-7]. Moreover, in many cases the extra disulfide bridge has been associated with greater antimicrobial potencies, especially at higher temperatures [7,8].

	In this study, we characterised the bacteriocin both genetically and phenotypically and assessed the *in vivo* performance of *Lb. plantarum* LMG P-26358 as an anti-listerial adjunct in the presence and absence of nisin-producing starters for the manufacture of laboratory and industrial scale cheeses. As *L. innocua* has previously been deemed a suitable biological indicator for *L. monocytogenes*[9] and the strain revealed similar sensitivity to the bacteriocin as eight other *L. monocytogenes* isolates tested, it was used as a pathogen surrogate throughout the study.

## Materials and methods

### Isolation of *Lactobacillus plantarum* LMG P-26358 and bacteriocin assays

*Lb. plantarum* LMG P-26358 was initially isolated from French artisanal cheese as follows: approximately 1g of cheese was homogenized in 9 mls of maximum recovery diluent (MRD), serially diluted and plated on MRS (de Man, Rogosa, Sharpe) agar (Difco Laboratories, Detroit, MI, USA) and grown at 30°C for 2-3 days. Colonies that developed were overlaid with ~10 ml of GM17 agar [M17 medium (Difco Laboratories) supplemented with 0.5% (w/v) glucose] inoculated at 0.25% with an overnight culture of *L. innocua* (DPC6579). The plates were incubated for another 18 h at 37°C and inspected for zones of inhibition of the overlaid culture. Colonies showing a clear zone of inhibition were sub-cultured into fresh MRS broth having first been removed from the agar overlay using a sterile scalpel blade. Pure cultures were obtained by streaking onto MRS agar. Bacteriocin assays and estimation of bacteriocin activity in activity units (au)/ml were performed by the agar well diffusion assay (WDA) as described by Ryan et al. [10].

The sensitivity of a strain to plantaricin 423 was scored according to the diameter of the zone of inhibition surrounding the well. Each assay was performed in triplicate. Other media used in the study included BHI (Brain-Heart Infusion) broth (Oxoid Ltd., Basingstoke, Hampshire, England), RCM (Re-inforced Clostridial Medium) (Merck, Darmstadt, Germany), LBS (Lactobacillus Selective) agar (Difco Laboratories), PCA (Total Plate Count) (Difco Laboratories), SLB (Sodium Lactate Broth) as described by Drinan and Cogan [11]. All strains were stocked in 50% glycerol at -20°C.

### Identification of *Lactobacillus plantarum* LMG P-26358

The antimicrobial strain was identified by analyzing the 16S rDNA sequence. Genomic DNA was isolated from 1.5 ml of overnight MRS broth culture using the method of Hoffman and Winston [12] with slight modification as described by Mills et al. [13]. The 16S rDNA primers CO1 (AGTTTGATCCTGGCTCAG) and CO2 (TACCTTGTTACGACTT) were used to amplify a product of ~1500 bp using an annealing temperature of 60°C. PCR amplification was performed in a Hybaid PCR express unit (Hybaid Ltd., Middlesex, UK) according to the manufacturer’s specifications using Biotaq DNA polymerase (BioTaq, Bioline Ltd., London, UK). The PCR product was purified using a Wizard SV Gel and PCR Clean-Up System (Promega, Madison, WI, USA) and sequenced with an automated DNA sequencer (Beckman Coulter Genomics, Hope End, Takeley, UK). The 16S rDNA gene sequence was analysed using BLAST [14] to identify the closest bacterial neighbour.

### Effect of proteinase K, pH and temperature on plantaricin 423 and stability of production

Proteinase K sensitivity was evaluated as follows: cell-free supernatant was harvested from 1 ml of overnight culture and exposed to a final concentration of 50 mg/ml proteinase K (Sigma-Aldrich, Poole Dorset, UK) and incubated for 2 hours at 37°C. The agar WDA was then performed against *L. innocua* DPC6579 with the proteinase K-treated sample and untreated cell-free supernatant as control. In order to determine the effect of pH on bacteriocin activity, the pH of cell-free supernatants was adjusted to pH values ranging from 1-10 using 1 M NaOH or 1 M HCl and incubated for 2 hours at 25°C before performing the agar WDA. In a separate experiment the effect of temperature on bacteriocin activity was assessed by incubating the cell-free supernatants at 40, 50, 60, 70, 80, 90 and 100°C for 30 min after which activity was assessed against *L. innocua*. Stability of bacteriocin production was assessed by sub-culturing *Lb. plantarum* LMG P-26358 (2%) in MRS broth twice a day for a 10 day period and performing the agar WDA against *L. innocua* each day. All experiments were performed in triplicate.

### HPLC purification and mass spectrometry

The bacteriocin was purified and the molecular mass determined as follows: 50 μl of stock culture was grown overnight at 37°C in 5 ml of MRS broth. Forty ml of MRS was inoculated at 1% from the overnight culture and incubated for 6-7 hours at 37°C and this was then used to provide a 1% inoculum for 2 L of MRS broth. Following overnight incubation at 37°C, the culture was centrifuged at 14,160 x g for 15 minutes and the supernatant discarded. Cells were mixed with 250 ml of 70% isopropanol, 0.1% trifluroacetic acid (TFA) and stirred for 3 hours at room temperature. Cells were re-centrifuged and the cell-free supernatant assayed for anti-listerial activity. The isopropanol was removed from the cell-free supernatant using a Buchi rotary evaporator (Buchi, Switzerland) and the resulting prep was passed through a 5 g, 20 ml Strata C18-E SPE column (Phenomenex, Cheshire, UK), the column was washed with 20 ml of 30% ethanol and bacteriocin was eluted with 20 ml of 70% isopropanol, 0.1% TFA. Isopropanol was removed from 20 ml of the bacteriocin-containing sample and this was then applied to a C12 Proteo reverse phase HPLC column running a 25-40% acetonitrile, 0.1% TFA gradient over 35 minutes. Two HPLC runs were typically done per 2 L prep. Mass spectrometry was performed on the anti-listerial fractions using an Axima TOF^2^ MALDI TOF mass spectrometer (Shimadzu Biotech, Manchester, UK). A 0.5-µl aliquot of matrix solution (Sinapinic acid, 10 mg/ml in 50% acetonitrile-0.1 % (v/v) TFA) was deposited onto the target and left for 5 seconds before being removed. The remaining solution was allowed air-dry and the sample solution was deposited onto the pre-coated sample spot. Matrix solution (0.5-µl) was added to the deposited sample and allowed air-dry. The sample was subsequently analysed in positive-ion linear mode. The purified peptide was resuspended in 0.1 M phosphate buffer (pH 6.8) and stored at -20°C. The activity in au/ml was determined by WDA as described previously [10].

### Identification of genes encoding plantaricin 423

N-terminal sequencing of the peptide was performed by Aberdeen Proteomics (Aberdeen University, Aberdeen, UK). Based on amino acid sequence similarity to plantaricin 423, primers designed to the structural gene, *plaA*, (423A5: AAATACTATGGTAATGGGG & 423A3: CATGGAAAGTGCTAATTA) as described by van Reenan et al. [15] and primers designed to the whole operon in this study (423F: ATGATGAAAAAAATTGAAAAA & 423R: CTTGATTATGAATTAACCGT) were used for PCR amplifications. DNA was amplified in a Hybaid PCR express unit. The Expand High Fidelity PCR system (Roche Diagnostics Ltd., East Sussex, UK) was used to amplify the products according to the Roche Diagnostics applications manual. The PCR product representing the whole operon was purified using the Wizard SV Gel and PCR Clean-Up System (Promega). The purified product was cloned into the TOPO XL PCR Cloning kit (Invitrogen, Paisley, UK). Clones were sequenced with an automated DNA sequencer (Beckman Coulter Genomics, UK) using the M13-Forward and Reverse priming sites on the pCR-XL-TOPO vector. Restriction enzymes were purchased from New England Biolabs (Hertfordshire, UK) and used according to manufacturer’s instructions. The sequence was annotated using ORF Finder (NCBI) and analysed using BLAST [14].

### Mode of action of plantaricin 423

The mode of action of plantaricin 423 against *L. innocua* was performed as described by Deraz et al. [16]. Briefly, a 1% inoculum of the culture was grown overnight. The following day the cells were harvested and resuspended in 0.1 M potassium phosphate buffer (pH 7.0). The sample was then divided into test and control and 2560 au/ml of a purified preparation of plantaricin 423 was added to the test. Both samples were incubated at 37°C for 8 hours. Optical density (600 nm) and cell numbers (colony forming units (cfu/)ml) were determined at 2 hour intervals. Each experiment was performed in triplicate and percentage killing was calculated according to the method of Deraz et al. [16] as follows:

% Killing = [((initial viable cells)-(final viable cells))**/**(initial viable cells)] x 100.

### Minimum inhibitory concentration (MIC) and specific activity of plantaricin 423

The MIC was determined according to the method of Gravesen et al. [17] and Ramnath et al. [18]. Briefly, 5 μl of a two-fold serial dilution of cell-free supernatant (2560 au/ml plantaricin 423) or purified peptide were spotted onto GM17 plates seeded with 0.25% *L. innocua*. The MIC was calculated as the minimal concentration that produced a visible zone after 18 hours at 37°C. The experiment was performed in triplicate. The specific activity was experimentally measured by generating a standard curve of bacteriocin concentration in mg/ml versus au/ml. The experiment was performed in triplicate with two different purified preparations of plantaricin 423. In both experiments the R^2^ value was above the 95% confidence level.

### Laboratory-scale cheese manufacture with *Lactobacillus plantarum* LMG P-26358

Cultures which included *L. lactis* DPC4268 (cheese starter), *L. lactis* CSK65 (nisin producer) and *Lb. plantarum* LMG P-26358 were inoculated into 1 L of whole milk heated to 32°C as follows: Vat 1 = 0.75 % *L. lactis* DPC4268 (cheese starter); Vat 2 = 0.75 % *L. lactis* DPC4268, 0.75 % *L. lactis* CSK65 (nisin producer); Vat 3 = 0.75 % *L. lactis* DPC4268, 0.75% *Lb. plantarum* LMG P-26358; Vat 4 = 0.75% *L. lactis* DPC4268, 0.5% *Lb. plantarum* LMG P-26358, 0.5% *L. lactis* CSK65. A streptomycin-resistant derivative of *L. innocua* (DPC6578) was added to each sample vat at a level of 10^3^ cfu/ml. Thirty min after inoculation Chymax rennet (Hansens, Little Island, Cork, Ireland) was added according to manufacturer’s instructions and the curd was cut at the appropriate time. The temperature was then elevated from 32°C to 38.5°C over a 30 min period. At pH 6.2, the whey was drained and the temperature was reduced to 32°C. When the curd reached pH 5.2, the curd was further drained and pressed into moulds overnight. The cheeses were then incubated at 20°C for 16 hours after which they were vacuum-packed and ripened at 12°C for 4 weeks. *L. innocua *DPC6578 was enumerated in each cheese on a weekly basis by homogenising 1 g of cheese in 2% sterile trisodium citrate and plating serial dilutions on GM17 agar containing streptomycin (500 μg/ml). *Lb. plantarum* LMG P-26358 was enumerated by plating on LBS agar. Each cheese trial was performed in triplicate.

### Industrial-scale Gouda cheese manufacture with *Lactobacillus plantarum* LMG P-26358

The starter cultures applied in the manufacture of industrial-scale Gouda cheese were produced by CSK Food Enrichment, The Netherlands. In each vat, *L. lactis* CSK 976 was used as the main starter culture. The vats were set up as follows: Vat 1 = *L. lactis* C976, *L. lactis* biovar *diacetylactis* C975 (nisin producer); Vat 2 = *L. lactis* C976, *Lb. plantarum* LMG P-26358; Vat 3 = *L. lactis* C976, *L. lactis* biovar *diacetylactis* C975, *Lb. plantarum* LMG P-26358; Vat 4 = *L. lactis* C976, *L. lactis* biovar *diacetylactis* C975, *Lb. plantarum* LMG P-26358. Vat 4 differed from Vat 3 in that it contained double the amount of *Lb. plantarum* LMG P-26358. Gouda cheese was manufactured at the pilot plant of Nizo Food Research Ede, The Netherlands. In each experiment 1500 L of milk was used per vat to produce 15 x 10 kg cheese wheels with 51% fat, 41 % moisture and 3% salt content. The milk was bactofugated at 68°C for 13 sec and pasteurized at 73°C for 13 sec, then the cheese milk was cooled down to 32°C. At this point nitrate (35% sol) and calcium choride (33% sol) were added in the amounts of 55 and 70 g, respectively, per 1500 L. The different starter/adjunct were added as frozen pellets that resulted in bacterial counts of 10^6^-10^8^ in fresh, unripened cheese. The amount of calf rennet, Ceska^®^-Lase 150 IMCU (CSK Food Enrichment, Ede, The Netherlands) added was 20 g per 1500 L cheese milk. After a coagulation time of 35 min, the curd was cut for 20 min and then 40 % of the whey was removed. The remaining lactose was washed out of the curd by adding hot water. The curd was further stirred for 33 min at a constant temperature of 35°C. After whey drainage, the cheese was pressed for 90 min and brined for 72 hours. The pH of the cheeses after one day was 5.2. The cheeses were coated with Ceska^®^-Coat during 18 weeks of ripening at 13°C. Cheese samples were evaluated for bioactive properties at different stages of ripening (day 1, day 14, weeks 6, 12 and 18).

### Microbiological analysis of the cheese

Samples extracted using a borer were collected in sterile bags. After discarding 1 cm of outer surface, the sample (app. 10 g) was homogenized with a 2% sterile trisodium citrate solution at 45°C (1:10 dilution). The homogenized samples were diluted in physiological water, and the plate count technique was used to determine the bacterial counts. *Lb. plantarum* counts were determined using commercially available MRS agar medium for selective enumeration and total *lactococci* counts were determined in commercially available M17 medium containing 1% lactose as the carbon source. Colonies were counted after anaerobic incubation for 3 days at 37°C or 30°C for lactobacilli or lactococci, respectively. All determinations were performed in duplicate.

The industrial cheeses were assayed for antimicrobial activity by homogenising 1 g of cheese in 9 mls of MRD to which 10^4^ cfu/ml of *L. innocua *DPC6578 was added from an overnight culture. A control was also set up which did not contain any cheese. The cheese/*Listeria* slurry was then incubated at room temperature over 24 hours and *Listeria* counts were determined at 5 and/or 24 hours by plating serial dilutions on GM17 containing streptomycin (500 μg/ml). A plug of each cheese was also overlaid with *L. innocua* to determine if plantaricin 423 was active.

### Detection of nisin and plantaricin 423 in industrial cheeses by MALDI-TOF mass spectrometry

Cheese samples were subjected to mass spectrometry to detect the presence of bacteriocins. Briefly, 20 ml of 70% isopropanol, 0.1% TFA was added to 1 g of cheese sample and mixed at room temperature for 3-4 hours. Cheese mixtures were centrifuged and 20 ml of 70% isopropanol, 0.1% TFA was added to the supernatant. The isopropanol was removed using rotary evaporation and suspensions were re-centrifuged to spin down particulate matter. The pH of the supernatant was adjusted to 4-5 by adding approximately 30 μl of 7.5 N NaOH and then passed through a 5 g 20 ml Phenomenex Strata C18-E SPE column, columns were washed with 20% ethanol and bacteriocins eluted with 70% isopropanol, 0.1% TFA. The isopropanol was removed from the 70% isopropanol, 0.1% TFA samples before applying to a semi prep C12 Proteo Jupiter RP-HPLC column running a 25-48% acetonitrile, 0.1% TFA gradient over 35 minutes. Eluent was monitored via UV at 214 nm. Fractions were collected at 1 min intervals and nisin- and plantaricin-containing fractions were analysed by MALDI-TOF mass spectrometry as previously described to confirm their presence.

### Spray-drying

A spray-dried powder of *Lb. plantarum* LMG P-26358 was generated by growing the strain to 8 L in 20% RSM with 0.5 % yeast extract and 0.2 g/l MnSO_4_.4H_2_O. The fermentate was concentrated to 40% total solids in a single-effect falling-film evaporator (Anhydro F1-Lab) before spray-drying. Concentrates were then dehydrated in a pilot-scale Anhydro spray drier (Model Lab 3) at an inlet temperature of 187°C and an air outlet temperature of ~ 85°C. The powder was assayed for viable cells by plating on PCA and LBS, and counts were compared across both sets of plates to determine the cfu/g of *Lb. plantarum* LMG P-26358. Anti-listerial activity was assessed by adding the powder at 1% (w/v), 5% (w/v), 10% (w/v) and 15% (w/v) to 10^4^ cfu/ml of *L. innocua *DPC6578 in GM17 broth. The culture/powder mix was incubated for 6 hours at 37°C and samples were removed every 2 hours to enumerate *Listeria* by selecting on GM17 with streptomycin (500 μg/ml). Each experiment was performed in triplicate.

## Results

### Isolation and identification of *Lactobacillus plantarum* LMG P-26358

*Lb. plantarum* was isolated from a soft French artisanal cheese due to its associated production of a large zone from the colony when overlaid with *L. innocua*. Following purification it was later identified as *Lb. plantarum* by 16S rDNA sequencing and designated *Lb. plantarum* LMG P-26358. To determine if the inhibitory activity was proteinaceous, the supernatant was treated with proteinase K and tested against *L. innocua* by WDA. The loss of activity confirmed that the antimicrobial substance produced by *Lb. plantarum* was indeed proteinaceous. The level of inhibitory activity in culture supernatant against *L. innocua* was determined to be 2560 au/ml (~7-8 mm zone) when measured by WDA. Sugar acidification profiles indicated that *Lb. plantarum* LMG P-26358 can grow efficiently on both glucose and lactose with generation times (GTs) of 100 mins and specific growth rates of 0.1814 and 0.1815, respectively. Maltose was the most efficient sugar for growth with a GT of 79 mins and specific growth rate of 0.2277, while the strain was unable to metabolise raffinose.

### Identification of the antimicrobial substance produced by *Lactobacillus plantarum* LMG P-26358 and characterisation of the genetic machinery

The antimicrobial substance was purified by HPLC and the molecular mass was determined to be 3929 Da using MALDI-TOF mass spectrometry. The peptide was then partially sequenced revealing the first 10 amino acids. These 10 amino acids were found to be identical to the class IIa bacteriocin plantaricin 423 which is produced by *Lb. plantarum* 423 and has a molecular mass of 3932.74 [15]. Based on the sequence of plantaricin 423 of *Lb. plantarum* 423 primers were generated to the bacteriocin structural gene and to the complete operon involved in the production of the bacteriocin. Both sets of primers generated PCR products of the correct size for the structural gene (150 bps) and the operon (~3582 bps) from *Lb. plantarum* LMG P-26358. DNA sequence analysis and annotation of the amplified operon revealed that the structural gene is identical to that of plantaricin 423 from *Lb. plantarum* 423. While 17 base pair differences were observed across the remainder of the operon, such changes could be considered silent in the majority of cases. The bacteriocin produced by *Lb. plantarum* LMG P-26358 was designated plantaricin 423.

### Effect of heat and pH on the activity of plantaricin 423 and stability of bacteriocin production by *Lactobacillus plantarum* LMG P-26358 over a 10 day period

Heat treatment of the bacteriocin at 40, 50, 60, 70, 80, 90 and 100°C for 30 minutes did not compromise its antimicrobial activity. Likewise, the activity of the bacteriocin was unaffected by pH as the bacteriocin continued to produce 2560 au/ml following incubation of the supernatant at pH values from 1-10 for 2 hours at 25°C. The producing strain, *Lb plantarum* LMG P-26358, was sub-cultured for 10 days and the supernatant was assayed for activity each day. The strain continued to produce equivalent potent activity against *L. innocua* on each day of the 10-day period.

### Spectrum of inhibition

The activity of plantaricin 423 was assayed against 34 indicator strains which included LAB starters such as *L. lactis* and *Lactobacillus* species as well as food spoilage and pathogenic bacteria such as *Bacillus subtilis* and *Salmonella typhi* and eight isolates of *L. monocytogenes*, six of which were previously isolated from smear-ripened cheeses and cheese plants. Apart from the expected inhibitory activity against *Listeria*, plantaricin 423 also inhibited *Enterococcus faecalis*, producing a 5 mm zone, and *Enterococcus faecium*, producing a 2 mm zone. The lack of inhibition against the starters tested in the study suggests that *Lb. plantarum* LMG P-26358 is a suitable adjunct for cheese manufacture.

### Mode of action of plantaricin 423

**Figure 1:**
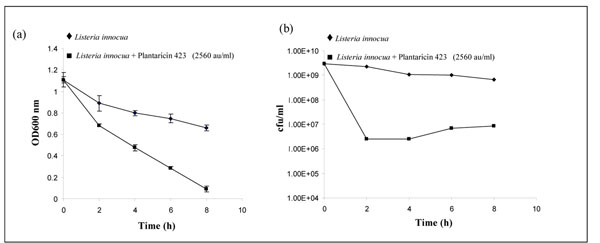
Mode of action of plantaricin 423 on *Listeria innocua*. Parameters tested were optical density (OD 600 nm) (a) and cell viability (cfu/ml) (b).

The effect of plantaricin 423 on *Listeria* was investigated to determine if it has a bactericidal or bacteriostatic mode of action. The purified peptide (resuspended in phosphate buffer) with an initial activity of 102,400 au/ml was added to cell suspensions at a final concentration of 2560 au/ml and growth was assessed by measuring the optical density and cell numbers over an eight hour period, and percentage killing was also calculated. Over the eight hour period, a marked decrease was observed in both optical density and cell viability for samples containing plantaricin 423, compared to controls (Figure 1). A three log reduction was recorded for cells in the presence of bacteriocin within the first 2 hours, corresponding to 99.9% killing and a 2 log reduction was observed after the eighth hour (99% killing). The dramatic decrease in optical density indicates that plantaricin 423 has a bactericidal effect on cells of *L. innocua* (Figure 1).

### Minimum inhibitory concentration and specific activity of plantaricin 423

**Figure 2:**
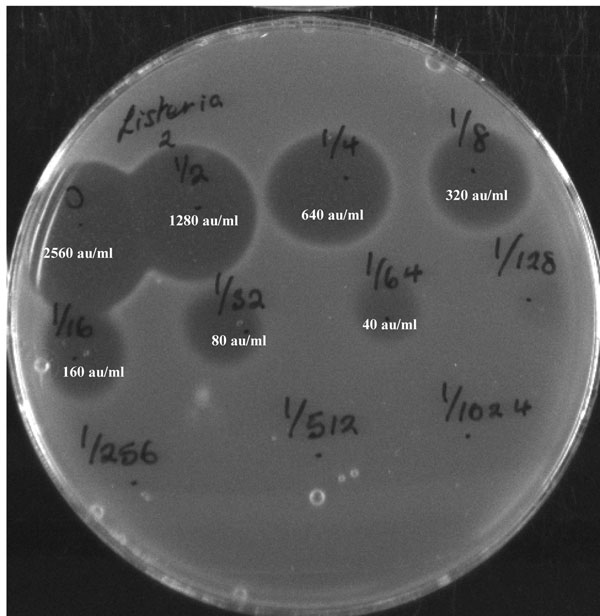
Minimum inhibitory concentration of plantaricin 423 against *Listeria innocua* defined as the minimal concentration showing a visible zone of inhibition.

The minimum inhibitory concentration (MIC) against *L. innocua* and eight isolates of *L. monocytogenes* was estimated to be 40 au/ml using purified plantaricin 423 or cell-free supernatant (Figure 2). A standard curve was generated to determine specific activity. The activity in mg/ml can thus be calculated using the equation y=10^-5^(X) where X is the activity expressed as au/ml and y is the activity expressed as mg/ml. Therefore, the MIC which was determined to be 40 au/ml is equivalent to 0.4 μg/ml (0.10173 μM).

### Laboratory-scale cheese production using *Lactobacillus plantarum* LMG P-26358 as an adjunct culture

*Lb. plantarum* LMG P-26358 was found to grow adequately in 10% RSM substituted with 0.1% yeast extract. Supplementation of the starter culture medium with 0.2 g/l MnSO_4_.4H_2_O was also shown to facilitate its growth. Combining the strain with the nisin producer *L. lactis* CSK 65 (produces 320 au/ml of nisin) eliminated the requirement for yeast extract in 10% RSM and the strain continued to produce a full zone of inhibition against *L. innocua* following overnight growth, although acidification and growth of *Lb. plantarum* LMG P-26358 was impeded in the presence of nisin in the first eight hours of growth. Studies suggested that at least 10^8^ cfu/ml of *Lb. plantarum* LMG P-26358 were required for optimal bacteriocin production. Laboratory-scale cheese spiked with 10^3^ cfu/ml of *L. innocua* was manufactured to test the anti-listerial capacity of *Lb. plantarum* LMG P-26358 in the presence and absence of the nisin producer *L. lactis* CSK65*.*

**Figure 3:**
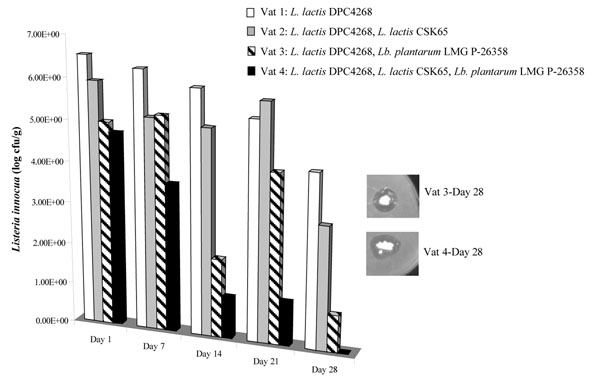
Counts of viable *Listeria innocua* cells in cheeses manufactured with the starter *Lactococcus lactis* DPC4268 (Vat 1) and accompanied with either *Lactococcus lactis* CSK 65 (Vat 2); *Lactobacillus plantarum* LMG P-26358 (Vat 3); *Lactococcus lactis* CSK 65 and *Lactobacillus plantarum* LMG P-26358 (Vat 4). Insert shows cheeses (day 28) from vats 3 and 4 overlaid with *Listeria innocua*.

Each cheese was ripened at 12°C for 4 weeks and *Listeria* and *Lb. plantarum* LMG P-26358 was enumerated each week of the ripening period (Figure 3). On day 28, a plug of each cheese was also overlaid with *L. innocua* to determine if the bacteriocin was still active. By day 28, *L. innocua* numbers were dramatically reduced in Vat 3 (*Lb. plantarum* LMG P-26358) as compared to Vats 1 and 2 and *Listeria* was not detected in Vat 4 (*Lb. plantarum* LMG P-26358 and nisin producer) suggesting that the combination of plantaricin 423 and nisin was more effective than plantaricin 423 alone. On days 1 and 28 after cheese manufacture *Lb. plantarum* LMG P-26358 was present at 10^8^ cfu/ml or more. Overlays of cheese plugs on day 28 indicated that the *Lb. plantarum* LMG P-26358-containing cheeses produced a zone against *L. innocua* (Figure 3).

### Industrial-scale Gouda cheese production using *Lactobacillus plantarum* LMG P-26358 culture

**Figure 4:**
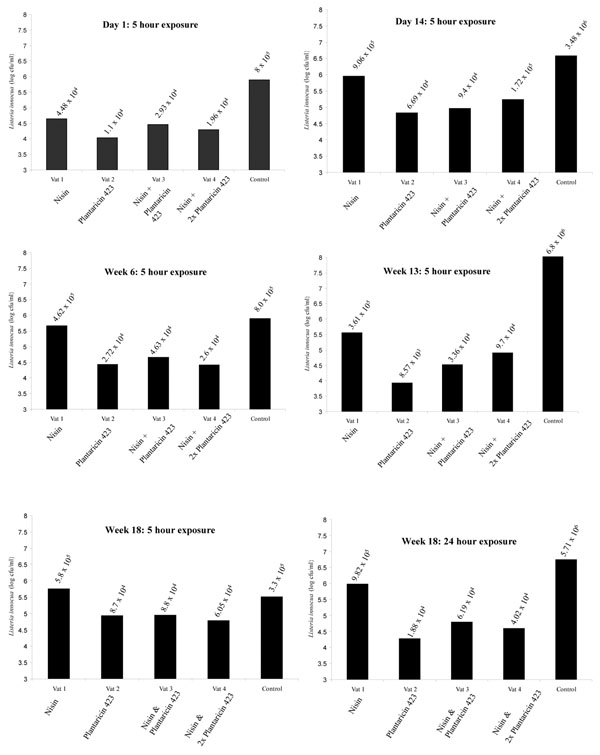
*Listeria innocua* counts following 5 or 24 hours of exposure to ~1g of industrial cheeses.

Industrial-scale Gouda cheese was manufactured at CSK Food Enrichment, The Netherlands. The cheeses were tested over an 18 week period for anti-listerial activity by generating cheese/*Listeria* slurries in MRD. The cheeses produced with *Lb. plantarum* LMG P-26358 were much more effective at controlling *Listeria* numbers than using the nisin producer alone (Figure 4). A slight antagonistic effect may have occurred between the nisin producer and *Lb. plantarum* LMG P-26358 as zones from cheese plugs of Vats 3 and 4 were always slightly smaller than zones from Vat 2 (results not shown). We have already observed that nisin does slow the growth of *Lb. plantarum* LMG P-26358 in the initial stages of growth. However, *Listeria* counts did not vary greatly between Vats 2, 3 and 4. Nisin and Plantaricin 423 were both detected in the appropriate cheeses by mass spectrometry over the 18 week ripening period (results not shown).

### Frequency of bacteriocin resistance development in *Listeria innocua*

The frequency of resistance development in *L. innocua* to 2560 au/ml plantaricin 423 (purified preparation) was calculated at 1.13 x 10^-3^. However, the frequency of resistance development against 320 au/ml nisin was lower at 2.16 x 10^-2^. Simultaneous exposure to plantaricin 423 and nisin at the above levels reduced the frequency of resistance development to 1.34 x 10^-4^. The resistant colonies were removed and tested for their sensitivity to plantaricin 423. However, all bacteriocin resistant strains (including nisin resistant and plantaricin 423 resistant) remained sensitive to 2560 au/ml suggesting that the cells were ‘tolerant’ rather than completely resistant. We did however isolate plantaricin 423 resistant derivatives of *L. innocua* by growing approximately 10^8^ exponential phase cells/ml overnight in the presence of a purified preparation of plantaricin 423 (2560 au/ml) in broth.

### Antimicrobial activity of spray-dried powder of *Lb. plantarum* LMG P-26358

**Figure 5:**
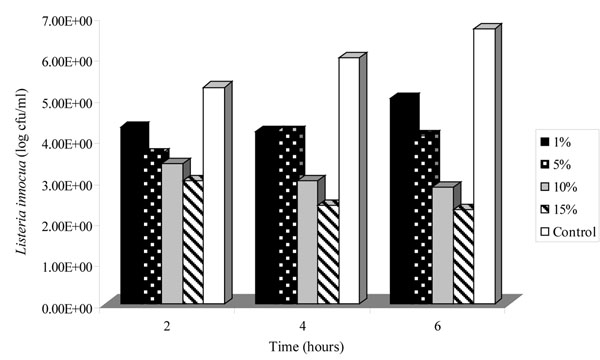
The effect of *Lactobacillus plantarum* LMG P-26358 spray-dried powder (1, 5, 10 and 15%) on the viability of *Listeria innocua*.

Preliminary results demonstrated that *Lb. plantarum* LMG P-26358 was suitable for spray-drying and the resulting powder was capable of inhibiting *L. innocua in vitro* demonstrating an avenue worthy of further investigation considering the wide range of applications for antimicrobial powders. A total of 8 L of an overnight fermentate of *Lb. plantarum* LMG P-26358 was spray-dried, yielding approximately 1 kg of powder containing 6.0 x 10^4^ viable cfu/g of *Lb. plantarum* LMG P-26358. The powder was tested against *L. innocua* at concentrations of 1, 5, 10 and 15%. All concentrations tested exhibited antimicrobial activity against the indicator strain (Figure 5). Indeed, at 15% the powder reduced *L*. *innocua* numbers by approximately 4 logs within 6 hours when compared with the control.

## Discussion

*Lb. plantarum* LMG P-26358 was isolated from a soft French artisanal cheese because of its ability to produce a bacteriocin against *L. innocua*. The bacteriocin structural peptide was found to be identical to the class IIa bacteriocin plantaricin 423 produced by *Lb. plantarum* 423, a strain previously isolated from sorghum beer by van Reenan et al. [19] although seventeen base pair differences were observed across the remainder of the operon. Plantaricin 423 produced by *Lb. plantarum* LMG P-26358 was found to have a bactericidal effect on *L. innocua* growth with a MIC of 40 au/ml, a MIC that was also observed against eight *L. monocytogenes* isolates. However, initial results suggested that at least 10^8^ cells of *Lb. plantarum* LMG P-26358 are required for optimal activity of the bacteriocin against *Listeria*. The bacteriocin was also found to inhibit enterococcal strains including *E. faecium* and *E. faecalis*. Dairy starters including *L. lactis* and various lactobacilli were insensitive to the bacteriocin.	

Resistance development in *Listeria* to class IIa bacteriocins is a well recognised phenomenon and is associated with the mannose phosphotransferase system, the receptor for class IIa bacteriocins in *Listeria*[20-22]. We observed that *L. innocua* developed resistance to plantaricin 423 (2560 au/ml) at a frequency of 10^-3^, and to nisin (320 au/ml) at a frequency of 10^-2^. When both nisin and plantaricin 423 were combined at the same concentrations the frequency of resistance development was reduced to 10^-4^. However, these experiments were performed using purified preparations of plantaricin 423 and nisin and the optimal conditions for *Listeria* growth. In the complex environment of cheese, the level of resistance development may be dramatically hindered by numerous hurdles including salt, temperature etc.

	Considering that bacteriocins can be extremely effective for food safety applications when used in combination with other hurdles as reviewed by Mills et al. [23] we evaluated the suitability of *Lb. plantarum* LMG P-26358 for use as an adjunct culture in cheese manufacture in terms of its anti-listerial capacity in conjunction with a nisin producer. The combination of nisin- and plantaricin- CSK producing cultures in laboratory cheeses (deliberately spiked with *L. innocua*) was found to dramatically reduce *Listeria* numbers. Indeed, by day 28 of ripening, the cheese manufactured with the nisin producer and *Lb. plantarum* LMG P-26358 had no viable *Listeria* cells, suggesting that the emergence of resistant mutants was not an issue in the cheese environment. Moreover, a plug of the cheese itself (< 1g) from day 28 of ripening produced a zone of inhibition against *L. innocua*. Importantly, *Lb. plantarum* numbers reached 10^8^ cfu/ml on the first day of cheese manufacture and existed at 10^9^ cfu/ml by day 28. Anti-listerial capacity was evaluated for industrial cheeses by adding 1 g of the cheese to *L. innocua* cells. The cheese slurry behaved as an anti-listerial ingredient and was shown to prevent the proliferation of *L. innocua*. Overall, the synergistic effect observed in nisin- and plantaricin 423-containing laboratory cheeses was not observed for the industrial-scale cheeses. However, this may be a reflection of the method of analysis which differed between both industrial and laboratory-scale cheeses. We could deduce that *Lb. plantarum* LMG P-26358 was much more effective at inhibiting *Listeria* than the nisin producer alone. Moreover, mass spectrometry confirmed the presence of nisin and plantaricin 423 in the appropriate cheeses over an 18 week period suggesting that the integrity of each bacteriocin remains intact in the complex cheese environment. Cheese plugs from the industrial cheeses containing *Lb. plantarum* LMG P-26358 continued to produce a zone of inhibition against *L. innocua* over the ripening period also.

A spray-dried powder was then generated from the fermentate of *Lb. plantarum* LMG P-26358. Preliminary results suggested that the powder exhibited significant anti-listerial activity during *in vitro *assays suggesting that such a bioactive ingredient could have beneficial applications as a food-safety ingredient in various dairy products including yogurts, cheeses and coatings.

In conclusion, we have demonstrated that *Lb. plantarum* LMG P-26358 isolated from a soft French artisanal cheese has potential as an anti-listerial adjunct starter in the manufacture of cheese using the biological indicator strain *L. innocua*. Moreover, the strain is compatible with nisin producers and dramatically increases the potency of nisin. However, although class IIa bacteriocins are renowned for their anti-listerial activity, the strain should be tested against a wide range of *L. monocytogenes* strains if it is to be used in food safety applications, preferably isolated from different regions. In addition, we have observed that the strain’s anti-listerial activity is less effective at levels ≤ 10^7^ cells/ml. Therefore, to ensure optimal protection we suggest that *Lb. plantarum* LMG P-26358 be used as an adjunct starter with nisin producers at levels of 10^8^ cell/ml or greater to improve the quality and safety of cheese products.

## Competing interests

The authors declare that they have no competing interests.
